# Supporting capacity for research on malaria in Africa

**DOI:** 10.1136/bmjgh-2018-000723

**Published:** 2018-04-12

**Authors:** Brian Greenwood, Oumar Gaye, Moses R Kamya, Gibson Kibiki, Victor Mwapasa, Kamija S Phiri, Harry Tagbor, Dianne Terlouw, Imelda Bates, Alister Craig, Pascal Magnussen, Thor G Theander, Amit Bhasin, Hazel McCullough, David Schellenberg

**Affiliations:** 1 London School of Hygiene & Tropical Medicine, London, UK; 2 Cheikh Anta Diop University, Dakar, Senegal; 3 Makerere University, Kampala, Uganda; 4 Kilimanjaro Christian Medical College, Moshi, Tanzania; 5 College of Medicine, University of Malawi, Blantyre, Malawi; 6 University of Health and Allied Sciences, Ho, Ghana; 7 Liverpool School of Tropical Medicine, Liverpool, UK; 8 University of Copenhagen and Copenhagen University Hospital, Copenhagen, Denmark

**Keywords:** malaria

## Abstract

Substantial progress has been made in the control of malaria in Africa but much remains to be done before malaria elimination on the continent can be achieved. Further progress can be made by enhancing uptake of existing control tools but, in high transmission areas, additional tools will be needed. Development and evaluation of these new tools will require a substantial cadre of African scientists well trained in many different disciplines. This paper describes the activities undertaken by the Malaria Capacity Development Consortium (MCDC) to support the careers of PhD students and postdoctoral fellows undertaking research on malaria at five African universities. A systematic assessment of constraints on PhD training and research support systems was undertaken at each partner African university at the beginning of the programme and many of these constraints were remedied. The success of the programme is shown by the fact that 18 of the 21 PhD students recruited to the programme completed their theses successfully within a 4-year period and that all 27 scientists recruited to the postdoctoral programme were still working in Africa on its completion. The work of the consortium will be continued through Career Development Groups established at each partner university and at an affiliated institution at the University of Nairobi and through the Developing Excellence in Leadership, Training and Science award from the Wellcome Trust made to one of the African partners. Lessons learnt during the MCDC programme may help the planning and execution of other research capacity development programmes in Africa.

**Summary box**

- As the complexity of malaria control increases and the number of countries approaching elimination expands, there is an increasing need for scientists trained in many different disciplines to guide the complicated work needed to achieve the final goal of elimination.
- Able, well-trained scientists will only continue to remain in a malaria endemic area if they are provided with continuing support on completion of their PhD and/or first postdoctoral fellowships.
- The MCDC programme has shown that providing a mentorship programme and access to small grants to support innovative ideas and training can be very successful in keeping scientists in a place where they can apply the skills that they have learnt during their training.
- Well-trained scientists will only stay in post in an endemic country if, in addition to personal support, their home institution provides an attractive environment for research; the Malaria Capacity Development Consortium (MCDC) programme has shown some of the ways in which this can be achieved.
- Supporting a young scientist through an effective PhD and first postdoctoral fellowship is relatively expensive if this is to be done well, but this investment will have been well worthwhile when these scientists become key research leaders and policy advisors in their own country as the Gates Malaria Partnership and MCDC programmes have shown can be the case.

## Introduction

There has been considerable progress in the control of malaria during the past two decades.^1^ This success has been achieved largely through the scaling up of established interventions and additional progress could be made by enhancing scale-up even further.^2^ However, in high transmission areas, progress has stalled, and additional, novel interventions are needed for these areas as well as for those approaching elimination.^3^ Development and evaluation of these new interventions will require endemic country scientists with skills covering a wide range of disciplines and competency in the many areas that make a sound clinical researcher.^4^ Such scientists are in short supply. A number of organisations are supporting programmes directed at filling this gap. This paper describes the ways in which the Malaria Capacity Development Consortium (MCDC) has contributed to this goal and discusses the lessons learnt during the course of this programme.

## The Gates Malaria Partnership (GMP)

MCDC succeeded the GMP, a collaboration between three northern and five African partners, established in 2000 with support from the Bill & Melinda Gates Foundation.^5^ This partnership supported six postdoctoral fellows and 36 African PhD students, recruited from across sub-Saharan Africa. GMP PhD students registered for their degree with one of the northern partners and benefited from both the technical and general support that this provided, although most of their research was conducted at an African partner institution. In addition, successful students were eligible to apply for a competitive 3-year first postdoctoral award. GMP was successful in producing a cohort of well-trained African scientists with a primary interest in malaria, many of whom have gone on to very successful careers in this field including a provice-chancellor for research, three deans of medical schools or schools of public health and three directors of research institutes. Their research has had a major impact on national and international policies for malaria control such as the successful introduction of seasonal malaria chemoprevention. However, this programme focused on the individual and not on the institutions in which the individuals supported by the programme were expected to work on completion of their fellowship.

## The MCDC

The MCDC included the same three northern partners as GMP, three GMP African partners and two new African universities (box 1). Major decisions on student selection, approval of research grant applications and allocation of resources were made by a steering committee on which each partner had a representative. The overall direction of the consortium’s programme was guided by an external advisory committee. The MCDC programme was supported by a small secretariat based at the London School of Hygiene & Tropical Medicine (LSHTM).

Box 1 **Partners in the Malaria Capacity Development Consortium (MCDC)**

**African**

- Cheikh Anta Diop University, Dakar, Senegal
- College of Medicine and the Malawi-Liverpool Wellcome Trust Clinical Research Programme, University of Malawi, Blantyre, Malawi
- Kilimanjaro Christian Medical College, Moshi, Tanzania*
- Kwame Nkrumah University of Science and Technology, Kumasi, Ghana*
- Makerere University, Kampala, Uganda

**Northern**

- London School of Hygiene & Tropical Medicine, London, UK
- Liverpool School of Tropical Medicine, Liverpool, UK
- University of Copenhagen, Copenhagen, Denmark

*Also partners in the Danida supported Building Stronger Universities programme with which MCDC collaborated.

MCDC set out to (A) support a new cohort of 20 PhD students based at five African universities, (B) provide continuing support for the scientists who had obtained a PhD with support from GMP and (C) support overall post graduate training in the five partner African universities (table 1).

**Table 1:** Malaria Capacity Development Consortium’s (MCDC) approach to research capacity development in African universities

Individuals: researchers and research programme		Institutions: research environment and systems
PhD programme	Postdoctoral programme	African partner institutions
**Funding:** stipend PhD research grants.	**Competitive funding:** re-entry grants/first postdoctoral awards initiative awards senior fellowship awards.	**Assessment: capacity and capability to deliver PhD programmes** baseline needs assessment – gaps identified, addressed and follow-up assessment.
**Training:** first year: 6-week research methodology course training/development visits to EU partners third year: leadership development programme.	**Training:** leadership development programme mentorship training: from mentee to mentor.	**Assessment: research management support systems** eight areas covering the entire research project cycle, recommendations and follow-up assessment.
**Support:** research supervisory teams: EU and African supervisors and advisors personal development planning.	**Support:** personal development planning (career development) formal mentorship programme.	**Support:** research supervision workshops training the trainer course personal development planning mentorship – mentors and mentees MCDC educational support visits.
**Other MCDC training and support:** data management statistics scientific writing. **Building networks and disseminating research:** attendance and presentation at annual MCDC meetings and international conferences (American Society of Tropical Medicine and Hygiene, Multilateral Initiative on Malaria and European Congress on Tropical Medicine and International Health).		**Sustainability beyond MCDC:** institutional Career Development Groups (CDGs) – to embed institution-led support, training and development for researchers within institutional practices, policies and processes.
**MCDC supported by:** external advisory committee: global leaders and senior scientists in malaria secretariat: director, deputy director, project manager, education advisor, administrator steering committee: principal investigators from all the consortium partners.		

### The MCDC PhD programme

Advertisements for PhD fellowships were made in each partner African country and short lists prepared by the local university, but a final decision on awards was made by the MCDC steering committee, an important concession by the partner universities. Twenty students (12 male and 8 female) were selected from 252 applicants from 20 African countries. Two students elected to undertake their PhD at a university in a country other than their own, one of whom completed his thesis successfully, but one returned to his home country for family reasons before completing his thesis. The topics of the students' theses are presented in online supplementary table S1.

10.1136/bmjgh-2018-000723.supp1 Supplementary data

MCDC PhD students registered at the African partner university and their primary supervisor was a member of that university. However, each student had an advisory committee that included a cosupervisor from one of the northern partner institutions and, in some cases, an additional advisor with special skills such as statistics. PhD students attended a 6-week residential Research Methodology Course at the College of Medicine, Blantyre, at the start of their fellowship. PhD students were eligible to join a personal development planning (PDP) programme (see below). Each student was provided with a grant of up to £40 000, which enabled him or her to undertake a relevant research project that led to a high quality thesis, publications in high impact journals and, in some cases, influenced the policy of a national malaria control programme. Short-term visits to a northern partner institution were supported if needed.

Seventeen of the 20 students (85%) successfully defended their thesis within a 4-year period; one student was recruited by an international organisation, another moved country and a third student did not complete her thesis. To date, over 50 publications in peer-reviewed journals have been published based on the research of the MCDC PhD students (online supplementary table S2).

### MCDC postdoctoral fellowships

A key objective of MCDC was to provide continuing support for the PhD students graduated through the GMP programme during their early postdoctoral career. To achieve this goal, the 37 postdoctoral fellows and successful PhD students previously supported through the GMP programme were invited to apply to become an ‘MCDC investigator’. This was a formal process, and applications were refereed. The 27 successful applicants signed a contract that set out the benefits that they would be entitled to as an MCDC investigator but also specified the contributions that they would be expected to make to the consortium, such as supervision of new PhD students and attendance at consortium meetings.

Benefits to which MCDC investigators were entitled included:

#### Research support

MCDC investigators were eligible to compete for three postdoctoral fellowships of up to $300 000 and three first postdoctoral awards of up to $150 000. Applications for these awards were evaluated by external referees, and a final decision on which projects should be supported was made by the MCDC steering committee. In addition, MCDC investigators were eligible to apply for small ‘innovation grants’ (maximum $50 000). These were established to allow collection of pilot data that would support a larger grant application, allow completion of a sound project that had gone over budget or run out of time or to fund other bridging activities. Seventeen awards were made, seven of which supported a successful bid for a major new grant.

#### Participation in a PDP programme

A PDP programme for PhD students and postdoctoral fellows was established during GMP, and 27 MCDC investigators elected to continue in this programme that provided a grant of $12 000 to MCDC investigators or $5000 to PhD students to spend on educational activities outside their formal scientific training. Activities for which these funds were used included attendance at a formal course outside the grantee’s main area of activity, visits to laboratories outside their host institution, purchase of small items of equipment, attendance at international conferences and subscription fees to professional organisations. Each grantee’s PDP programme was monitored by MCDC’s educational advisor, and regular reports, including financial reports, were required. A review of the PDP programme in 2013 showed that grantees were turning increasingly to institutions in Africa to provide the extra skills that they were seeking. By the end of 2015, the five MCDC partner universities had embedded training in general skills for researchers within their institutional policies and practices.

#### Participation in a mentoring programme

A formal mentorship programme was established in January 2011. The 27 MCDC investigators who joined the scheme were invited to choose their mentor, and the suitability of this person was assessed by the MCDC secretariat. If an investigator had difficulty in identifying a mentor, the MCDC secretariat helped in the selection of a well-qualified person. Senior scientists tended to choose a research leader outside Africa who had managerial experience and international connections, while more junior scientists preferred a mentor based in their own or in an affiliated African institution. The mentorship programme was ‘light touch’ with the responsibility for the interaction lying with the mentor and the mentee rather than being imposed by the MCDC secretariat. Nevertheless, both mentors and mentees were required to sign a formal contract that set out their individual responsibilities. Responsibility for initiating and maintaining contact was placed on the mentee, while the mentor was required to have at least one meeting, ideally face to face, with his/her mentee each year. The programme was reviewed annually and used indicators to assess process, outcome and impact. Frequency, mode and initiation of contact were used to measure process and engagement. Results from the meeting discussions and the key milestones in the mentoring relationship (building rapport, establishing direction and purpose and measuring progress) were used to measure outcome and impact of the mentoring support. At the last review, 98% of the mentees interviewed considered that the mentoring programme had helped their career, and 85% of the mentors were happy with the progress of their mentee. All African partner institutions, and an MCDC affiliated institution, have now established their own mentorship programmes. Materials that were developed to support MCDC mentors and mentees and have been used by several other capacity development programmes; they are available on the MCDC website.^6^

#### Attendance at general training programmes

MCDC investigators were eligible to apply for a variety of training courses supported by MCDC, such as courses for PhD supervisors, training of PhD supervisors and a leadership training course.

#### Attendance at MCDC consortium meetings

MCDC investigators were expected to attend MCDC consortium meetings (see below).

### Strengthening overall research capacity development at the partner African universities

A number of activities were undertaken to strengthen the overall research capacity of the five African partner universities. These included:

#### Undertaking a baseline research capacity needs assessment

At the start of the programme, a baseline needs assessment of the challenges to research capacity development at each African partner university was conducted by a team from the Liverpool School of Tropical Medicine (LSTM) and Kwame Nkrumah University of Science and Technology using a defined plan.^7^ The team identified a number of challenges that were common to each university, such as a lack of information on university procedures, limited office and laboratory space for PhD students and research fellows, poor internet connectivity and library facilities and limited PhD supervision, as well as individual constraints. Each university was given a copy of the report on their institution and asked to prepare a plan as to how these weaknesses could be addressed. MCDC was able to provide small grants to support some of the activities outlined in each response, for example, preparation of a PhD handbook and an electronic PhD supervisor log. A follow-up review undertaken 3 years after the initial survey established that many of the recommendations made had been met. For example, a doctoral students’ handbook had been produced, and slow internet problems were remedied by donors cofunding a faster broadband connection.

#### Undertaking a research management support systems assessment

A second, more ambitious, review of overall research management was undertaken in September/October 2014 at four of the five partner universities.^8^ Since there was no pre-existing benchmark against which to assess the institutions’ research management support systems, a literature review was used to identify and describe all the components that make up such systems and to list global best practice for items within each component. This ‘benchmark’ was used to guide the development of data collection tools that comprised an online survey, and guides for conducting interviews, for reviewing institutions documents and for observing facilities. The initial survey, undertaken online by a team from LSTM and partner African universities, was followed by an on-site visit. Interviews were held with a wide range of staff ranging from students to senior university administrators. The review covered areas such as university research strategies and policies, institutional support in grant preparation, human resource management, external promotion of the university’s research and interactions with national government. Common gaps in the research support management systems included a lack of research strategies, inability to e-track research projects and inadequate quality checks for proposal submissions and contracts. Confidential recommendations were made as to how these might be resolved based on discussions held at the end of each site visit with stakeholders at the institutions. Contact was kept with each university through Skype calls during the following 18 months, and a final review of progress was undertaken in May 2016. Improvements were noted in a number of areas, in particular support for research grant development, with creation of a research grants office in two universities. However, universities still found it challenging to establish an overall research strategy for their institution and to actively promote uptake of research findings. Partner institutions found the evaluation process very helpful for strategic planning and for justifying and targeting resources towards key research capacity gaps. Using the same evidence-informed benchmark for all institutions enabled comparisons to be made and common gaps identified. Such common gaps are likely to be generalisable beyond the institutions in this study and would potentially be ‘smart’ investments for governments and health research funders.

#### Training courses and workshops

Training courses and workshops provided by MCDC included courses for PhD supervisors, trainers of PhD supervisors and mentors and a 1-year leadership course, led by Quilibra Consulting,^9^ for 13 PhD students and postdoctoral fellows identified as having leadership potential. This innovative course comprised four, 3-day workshops together with one-to-one coaching before and after each workshop. In addition, course participants were provided with a short placement with the management team of a successful commercial or academic institution. The course introduced participants to leadership issues such as time management, setting priorities and staff management. A formal review at the end of the course elicited very high scores from the participants, and there was universal recognition that, despite the time commitments, the course had led to profound insights that changed the attendees’ outlook both within and beyond their work environment.

#### Career Development Groups (CDGs)

To ensure the sustainability of the career development activities undertaken by the MCDC secretariat in London, CDGs were established in each African partner university and at The Centre for Biotechnology and Bioinformatics, University of Nairobi, Kenya. The aim of these groups is to embed sustainable research training and career development support within the practices of their institutions. These groups are led by a strategic lead and a group leader with responsibility for one or more aspects of career development such as mentoring, PDP or research supervision. Organisation of the CDGs and planning of their activities was helped by site visits and three meetings that allowed sharing of experience, expertise and resources across the partner universities.

#### Malaria centres

The African partner universities were encouraged to establish ‘Malaria Centres’ that brought together research groups from different parts of the university or affiliated institutions with an interest in malaria, and funds were provided by MCDC to initiate these activities; three of the five partner universities now have a malaria coordinating group.

#### Internships

An innovative, 1-year malaria research internship programme was piloted by the Malawi-Liverpool Wellcome Trust (MLW) Clinical Research programme to attract talented young scientists to malaria research. Candidates received a stipend and some PDP funding and were linked to an existing project. The main objective of these internships was to support the candidate to become more competitive, provide leadership training and support them in preparing a grant application. All 10 interns secured further personal funding on completion of their internship. Based on these positive results, both the College of Medicine, Blantyre and the MLW programme have included internships as part of their institutional postgraduate training programmes.

### MCDC consortium meetings

Four general meetings of the consortium were held to which PhD students, their supervisors, MCDC investigators, members of the MCDC steering committee, members of the MCDC external advisory committee and visiting lecturers were invited. These meetings were important in strengthening personal relationships within the consortium. Two consortium meetings that were held immediately prior to meetings of the American Society of Tropical Medicine and Hygiene (ASTMH) provided an opportunity for the MCDC PhD students and postdoctoral fellows to rehearse their presentation at the main meeting. Two MCDC PhD students won ASTMH Young Investigator awards. Consortium meetings also provided an opportunity for workshops on PDP, mentoring, data management and grant writing. A statistician and a data manager held open ‘surgeries’ during these meetings to provide advice to anyone who needed help in these areas.

## Conclusions and lessons learnt

The MCDC programme has been successful in meeting its immediate objectives and personal experiences of how it has helped individual African scientists can be seen in a video available on the consortium’s website.^6^ It has shown that African universities can produce PhD graduates whose research on malaria meets the highest standards, leads to high impact publications and can influence national malaria control policy.^10 11^ Recruiting a group of PhD students at approximately the same time to work on a common theme provided a cohort of interactive students who were able to support each other’s research and who had an impact on the research capacity activities of their institution. This proved to be a successful approach that might be adopted beneficially by other research capacity development programmes. A further objective of MCDC was encouraging the PhD students supported by GMP to remain in sub-Saharan Africa and to use the skills acquired during their fellowship to support malaria control or a related activity on the continent. This goal was also achieved, and all 27 scientists who joined this part of the MCDC programme as an MCDC investigator are still working in sub-Saharan Africa at the time of preparation of this is paper: 15 are based in universities, 7 in research institutes and 5 are working with government, non-governmental or international organisations and nearly all are involved in some kind of research. Key to achieving this goal was provision of small ‘initiative’ grants, which could be used in a variety of ways to help in sustaining their career, and establishment of a formal mentorship programme.

The conduct of an initial baseline needs assessment to identify the factors that might hinder the progress of PhD students and postdoctoral fellows at the African partner universities using a structured approach and subsequent monitoring of progress in addressing the challenges identified proved very valuable.^7 12^ Conducting a similar exercise at the start of any research capacity development programme is strongly recommended. Regular monitoring and evaluation of the general research capacity activities of the consortium, for example the mentorship programme, was undertaken by the consortium’s educational advisor and occasionally by external groups,^13^ and results from these activities were used to modify various components of the programme.

The MCDC programme could be criticised on the grounds that the financial support that it provided to its PhD students and postdoctoral fellows was possible only because of generous external funding and not a practical approach for most sub-Saharan African universities. Other less ambitious and expensive research capacity development programmes can be effective.^14^ However, our experience is that underfunding of a PhD fellowship often produces research of doubtful value and a graduate who lacks the skills or incentive to become a successful scientist, is liable to move out of science and whose training is thus a waste of funds. However, whether the financial investment provided by MCDC in a group of talented young African scientists was a sound one will become apparent only later in their careers when they achieve positions of responsibility. Funders of research capacity development programmes need to recognise that the full impact of programmes such as MCDC may take many years to become apparent, as shown by the GMP programme with its longer period of follow-up, and it is consequently important that the African partner institutions keep track of their graduates.

Areas in which MCDC did not achieve as much success as had been hoped, and which may need particular attention in future programmes, include the difficulties faced by both PhD students and postdoctoral fellows in setting up a research programme quickly in the context of an overstretched and slow university administrative system that also resulted, in many cases, in a cumbersome process of PhD submission and examination. There has been some cross-country collaboration between students and postdocs on completion of their fellowships, but this has been less than had been envisaged would be the case, and encouraging collaborations of this kind could be a particular goal for future research capacity development programmes.

The transition from GMP to MCDC involved an increasing role for the African partners in the consortium. However, overall administration of the MCDC programme remained with LSHTM. Thus, the next stage in the evolution to a fully independent African research capacity development programme required the transfer of the running of the programme to an African university and this has now been achieved. In 2015, the Wellcome Trust established a new research capacity development programme (Developing Excellence in Leadership, Training and Science (DELTAS)) and one of the partners in MCDC, University Cheikh Anta Diop, Dakar, Senegal, was successful in obtaining a highly competitive DELTAS grant. This new programme that is supported by three of the northern partners involved in the GMP and MCDC programmes and that will, like other research capacity development programmes in low-income countries, benefit from the experience of these previous programmes, is now underway providing a further opportunity to define the optimum ways for training the high-quality scientists who will be needed for many years to come if malaria is to be eliminated from Africa.
