# Supplementary material for: Supporting capacity for research on malaria in Africa
Source: BMJ Glob Health. 2018 Apr 12;3(2):e000723. doi: 10.1136/bmjgh-2018-000723 (PMC5898295; doi:10.1136/bmjgh-2018-000723)
Supplement: Supplementary data [file bmjgh-2018-000723supp001.pdf]

SUPPLEMENTARY TABLES

Table S1. Topics of the research projects of 20 MCDC PhD students.

University	Project Title	Completed
**Makerere University, Kampala, Uganda**	Sentinel surveillance to measure malaria burden	No
	Schools based ITN distribution	Yes
	Over treatment of malaria, RDTs	No
	Plasmodium-helminth associations and effects on malaria in infancy/childhood	Yes
**College of Medicine, University of Malawi, Blantyre, Malawi (COM)**	Immune responses to DBL domains	Yes
	A cluster-randomized trial of Intermittent Preventive treatment and Home Management of malaria in children in Malawi	Yes
	Malaria incidence in HIV exposed children after stopping Cotrimoxazole-Prophylaxis for HIV Opportunistic Infections	Yes
**Cheikh Anta Diop University, Dakar, Senegal (UCAD)**	IPTi : SP resistance markers	Yes
	Community based management of malaria	Yes
	Cytogenetic study of An Funestus and insecticide resistance	Yes
	sIPT with Amodiaquine/SP	Yes
**Kilimanjaro Christian Medical College, Moshi, Tanzania (KCMC)**	Anopheles diversity and insecticide resistance	Yes
	Behavioural responses of mosquitoes to insecticides/repellents	Yes
	Insecticide resistance	Yes
	Cluster randomised trial	Yes
**Kwame Nkrumah University of Science and Technology, Kumasi, Ghana (KNUST)**	Larval Habitat Characterisation	Yes
	IPTp - MQAS study	Yes
	IPTp Edipemiology	No
	An evaluation of the Intermittent Preventive Treatment of malaria in pregnancy (IPTp) programme in the Bosomtwe district of Ghana	Yes
	A comparison of methods of Assessment of Malaria attributable deaths in a rural district in Ghana	Yes

Table S2. Publications by MCDC PhD students based on work conducted during their PhD. Citation indexes are obtained from Google Scholar.

2010

1. **Nankabirwa J,** Cundill B, Clarke S, Kabatereine N, Rosenthal PJ, Dorsey G, Brooker S, Staedke SG. Efficacy, safety, and tolerability of three regimens for prevention of malaria: a randomized, placebo-controlled trial in Ugandan schoolchildren. PLoS One 2010;19:e13438. doi: 10.1371/journal.pone.0013438. PubMed PMID: 20976051; PubMed Central PMCID: PMC2957410.
   Citation index: 28

2011

2. **Kabula B**, Derua YA, Tungui P, Massue DJ, Sambu E, Stanley G, Mosha FW, Kisinza WN. Malaria entomological profile in Tanzania from 1950 to 2010: a review of mosquito distribution, vectorial capacity and insecticide resistance. Tanzan J Health Res 2011;13 Suppl 1:319-31.
   Citation index: not found.
3. Sindato C**, Kabula B**, Mbilu TJ, Manga C, Tungu P, Kazimoto JP, Kibonai SN, Kisinza WN, Magesa SM. Resting behaviour of Anopheles gambiae s.l. and its implication on malaria transmission in Uyui District, western Tanzania. Tanzan J Health Res 2011;13:122-5. PubMed PMID: 26592058
   Citation Index: 10
4. **Tine RC,** Faye B, Ndour CT, Ndiaye JL, Ndiaye M, Bassene C, Magnussen P, Bygbjerg IC, Sylla K, Ndour JD, Gaye O. Impact of combining intermittent preventive treatment with home management of malaria in children less than 10 years in a rural area of Senegal: a cluster randomized trial. Malar J 2011; 10:358. doi: 10.1186/1475-2875-10-358. PubMed PMID: 22166001; PubMed Central PMCID: PMC3286438.
   Citation index: 23

2012

5. **Kabula B**, Tungu P, Matowo J, Kitau J, Mweya C, Emidi B, Masue D, Sindato C, Malima R, Minja J, Msangi S, Njau R, Mosha F, Magesa S, Kisinza W. Susceptibility status of malaria vectors to insecticides commonly used for malaria control in Tanzania. Trop Med Int Health 2012;17:742-50. doi: 10.1111/j.1365-3156.2012.02986.x. Epub 2012 Apr 23. PubMed PMID: 22519840.
   Citation index: 22
6. **Kitau J,** Oxborough RM, Tungu PK, Matowo J, Malima RC, Magesa SM, Bruce J, Mosha FW, Rowland MW. Species shifts in the Anopheles gambiae complex: do LLINs successfully control Anopheles arabiensis? PLoS One 2012;7:e31481. doi: 10.1371/journal.pone.0031481. Epub 2012 Mar 16. PubMed PMID: 22438864; PubMed Central PMCID: PMC3306310.
   Citation index: 100
7. **Ndiaye M**, Faye B, Tine R, Ndiaye JL, Lo A, Abiola A, Dieng Y, Ndiaye D, Hallett R, Alifrangis M, Gaye O. Assessment of the molecular marker of Plasmodium falciparum chloroquine resistance (Pfcrt) in Senegal after several years of chloroquine withdrawal. Am J Trop Med Hyg 2012;87:640-5. doi: 10.4269/ajtmh.2012.11-0709. Epub 2012 Aug 27. PubMed PMID: 22927495; PubMed Central PMCID: PMC3516312.
   Citation index: 33

8. **Ndibazza J,** Mpairwe H, Webb EL, Mawa PA, Nampijja M, Muhangi L, Kihembo M, Lule SA, Rutebarika D, Apule B, Akello F, Akurut H, Oduru G, Naniima P, Kizito D, Kizza M, Kizindo R, Tweyongere R, Alcock KJ, Muwanga M, Elliott AM. Impact of anthelminthic treatment in pregnancy and childhood on immunisations, infections and eczema in childhood: a randomised controlled trial. PLoS One 2012;7:e50325. doi: 10.1371/journal.pone.0050325. Epub 2012 Dec 7. PubMed PMID: 23236367; PubMed Central PMCID: PMC3517620.
   Citation index: 46
9. **Tine RC, Ndiaye M**, Hansson HH, Ndour CT, Faye B, Alifrangis M, Sylla K, Ndiaye JL, Magnussen P, Bygbjerg IC, Gaye O. The association between malaria parasitaemia, erythrocyte polymorphisms, malnutrition and anaemia in children less than 10 years in Senegal: a case control study. BMC Res Notes 2012;5:565. doi: 10.1186/1756-0500-5-565. PubMed PMID: 23057857; PubMed Central PMCID: PMC3520821.
   Citation index: 24

2013

10. Jones CM, Haji KA, Khatib BO, Bagi J, Mcha J, Devine GJ, Daley M, **Kabula B**, Ali AS, Majambere S, Ranson H. The dynamics of pyrethroid resistance in Anopheles arabiensis from Zanzibar and an assessment of the underlying genetic basis. Parasit Vectors 2013;6: 43. doi: 10.1186/1756-3305-6-343. PubMed PMID:24314005; PubMed Central PMCID: PMC3895773.
    Citation index: 41
11. **Mosha JF**, Sturrock HJ, Greenhouse B, Greenwood B, Sutherland CJ, Gadalla N, Atwal S, Drakeley C, Kibiki G, Bousema T, Chandramohan D, Gosling R. Epidemiology of subpatent Plasmodium falciparum infection: implications for detection of hotspots with imperfect diagnostics. Malar J 2013;12:221. doi: 10.1186/1475-2875-12-221. PubMed PMID: 23815811; PubMed Central PMCID: PMC3701503.
    Citation index: 67
12. **Ndibazza J,** Webb EL, Lule S, Mpairwe H, Akello M, Oduru G, Kizza M, Akurut H, Muhangi L, Magnussen P, Vennervald B, Elliott A. Associations Between Maternal Helminth and Malaria Infections in Pregnancy and Clinical Malaria in the Offspring: A Birth Cohort in Entebbe, Uganda. J Infect Dis 2013;208:2007-16. doi: 10.1093/infdis/jit397. Epub 2013 Jul 31. PubMed PMID: 23904293; PubMed Central PMCID: PMC3836463.
    Citation index: 17
13. **Nankabirwa J**, Wandera B, Kiwanuka N, Staedke SG, Kamya MR, Brooker SJ. Asymptomatic Plasmodium infection and cognition among primary schoolchildren in a high malaria transmission setting in Uganda. Am J Trop Med Hyg 2013;88:1102-8. doi: 10.4269/ajtmh.12-0633. Epub 2013 Apr 15. PubMed PMID: 23589533; PubMed Central PMCID: PMC3752809.
    Citation index: 25
14. **Ndiaye M**, Tine R, Faye B, Ndiaye JL, Lo AC, Sylla K, Abiola A, Dieng Y, Ndiaye D, Hallett R, Gaye O, Alifrangis M. Selection of antimalarial drug resistance after intermittent preventive treatment of infants and children (IPTi/c) in Senegal. Am J Trop Med Hyg 2013;88:1124-9. doi: 10.4269/ajtmh.12-0739. Epub 2013 Apr 15. PubMed PMID: 23589534; PubMed Central PMCID: PMC3752812.
    Citation index: 15

15. **Ndiaye Y**, Ndiaye JL, Cisse B, Blanas D, Bassene J, Manga IA, Ndiath M, Faye SL, Bocoum M, Ndiaye M, Thior PM, Sene D, Milligan P, Gaye O, Schellenberg D. Community case management in malaria: review and perspectives after four years of operational experience in Saraya district, south-east Senegal. Malar J 2013;12:240. doi: 10.1186/1475-2875-12-240. PubMed PMID: 23849053; PubMed Central PMCID: PMC3716525.
Citation index: 18
16. Protopopoff N, Matowo J, Malima R, Kavishe R, Kaaya R, Wright A, West PA, Kleinschmidt I, Kisinza W, **Mosha FW,** Rowland M. High level of resistance in the mosquito Anopheles gambiae to pyrethroid insecticides and reduced susceptibility to bendiocarb in north-western Tanzania. Malar J 2013;12:149. doi: 10.1186/1475-2875-12-149. PubMed PMID: 23638757; PubMed Central PMCID: PMC3655935.
Citation index: 87
17. **Tine RC,** Ndiaye P, Ndour CT, Faye B, Ndiaye JL, Sylla K, Ndiaye M, Cisse B, Sow D, Magnussen P, Bygbjerg IC, Gaye O. Acceptability by community health workers in Senegal of combining community case management of malaria and seasonal malaria chemoprevention. Malar J 2013;12:467. doi: 10.1186/1475-2875-12-467. PubMed PMID: 24378018; PubMed Central PMCID: PMC3893441.
Citation index: 7
18. **Tine RC,** Ndour CT, Faye B, Cairns M, Sylla K, Ndiaye M, Ndiaye JL, Sow D, Cisse B, Magnussen P, Bygbjerg IC, Gaye O. Feasibility, safety and effectiveness of combining home based malaria management and seasonal malaria chemoprevention in children less than 10 years in Senegal: a cluster-randomised trial. Trans R Soc Trop Med Hyg 2014;108:13-21. doi: 10.1093/trstmh/trt103. Epub 2013 Dec 1. PubMed PMID: 24296325.
Citation index: 13

2014

19. Assefa SA, Preston MD, Campino S, **Ocholla H,** Sutherland CJ, Clark TG. estMOI: estimating multiplicity of infection using parasite deep sequencing data. Bioinformatics 2014;30:1292-4. doi: 10.1093/bioinformatics/btu005. Epub 2014 Jan 17. PubMed PMID: 24443379; PubMed Central PMCID: PMC3998131.
Citation index: 21
20. **Kabula B,** Tungu P, Malima R, Rowland M, Minja J, Wililo R, Ramsan M, McElroy PD, Kafuko J, Kulkarni M, Protopopoff N, Magesa S, Mosha F, Kisinza W. Distribution and spread of pyrethroid and DDT resistance among the Anopheles gambiae complex in Tanzania. Med Vet Entomol 2014;28:244-52. doi: 10.1111/mve.12036. Epub 2013 Nov 5. PubMed PMID: 24192019.
Citation index: 14
21. **Kabula B,** Kisinza W, Tungu P, Ndege C, Batengana B, Kollo D, Malima R, Kafuko J, Mohamed M, Magesa S. Co-occurrence and distribution of East (L1014S) and West (L1014F) African knock-down resistance in Anopheles gambiae sensu lato population of Tanzania. Trop Med Int Health 2014;19:331-41. doi: 10.1111/tmi.12248. Epub 2014 Jan 6. PubMed PMID: 24386946; PubMed Central PMCID: PMC4190685.
Citation index: 8

22. **Kitau J,** Oxborough R, Matowo J, Mosha F, Magesa SM, Rowland M. Indoor residual spraying with microencapsulated DEET repellent (N, N-diethyl-m-toluamide) for control of Anopheles arabiensis and Culex quinquefasciatus. Parasit Vectors 2014;7:446. doi: 10.1186/1756-3305-7-446. PubMed PMID: 25249021; PubMed Central PMCID: PMC4261282.
Citation index: 11
23. **Kitau J,** Oxborough R, Kaye A, Chen-Hussey V, Isaacs E, Matowo J, Kaur H, Magesa SM, Mosha F, Rowland M, Logan J. Laboratory and experimental hut evaluation of a long-lasting insecticide treated blanket for protection against mosquitoes. Parasit Vectors 2014;7:129. doi: 10.1186/1756-3305-7-129. PubMed PMID: 24679345; PubMed Central PMCID: PMC3973002.
Citation index: 11
24. **Matowo J,** Jones CM, Kabula B, Ranson H, Steen K, Mosha F, Rowland M, Weetman D. Genetic basis of pyrethroid resistance in a population of Anopheles arabiensis, the primary malaria vector in Lower Moshi, north-eastern Tanzania. Parasit Vectors 2014;7:274. doi: 10.1186/1756-3305-7-274. PubMed PMID: 24946780; PubMed Central PMCID: PMC4082164.
Citation index: 17
25. **Matowo, J.,** Kitau, J., Kabula, B., Kavishe, R. A., Oxborough, R. M., Kaaya, R., Francis, P., Chambo, A., Mosha, F. W. and Rowland M. W. Dynamics of insecticide resistance and the frequency of kdr mutation in the primary malaria vector Anopheles arabiensis in rural villages of Lower Moshi, North Eastern Tanzania. J. Parasitol Vector Biol 2014;6:31-41. DOI: 10.5897/JPVB2013.0143.
Citation Index: 9
26. **Ndiaye M**, FayeB, Tine R, Ndiaye JL, Sylla K, Sow D, Lo AC, Abiola A, Dieng Y Cisse B, Gaye O. Genetic analysis of erythrocyte binding antigen 175 (EBA-175), apical membrane antigen (AMA-1) and merozoite surface protein 3 (MSP-3) allelic types in Senegalese P. falciparum isolates. Malar Chemoth Cont Elimination 2014;3:113.doi :10.4172/2090-2778.1000113.
Citation index: 0
27. **Ndiaye M,** Ndiaye JL, Tine R, Sylla K, Faye B, Diouf I, Sow D, Lo AC, Abiola A, Dieng Y, Gaye O. Profile of cytokines associated with protection against malaria episodes during pregnancy in hypo-endemic area in Senegal. Bull Soc Pathol Exot 2014;107:159-164.
DOI 10.1007/s13149-014-0365-6
Citation index: 0
28. **Mbeye NM**, ter Kuile FO, Davies MA, Phiri KS, Egger M, Wandeler G; IeDEA-Southern Africa. Cotrimoxazole prophylactic treatment prevents malaria in children in sub-Saharan Africa: systematic review and meta-analysis. Trop Med Int Health 2014 19:1057-67. doi: 10.1111/tmi.12352. Epub 2014 Jul 8. Review. PubMed PMID: 25039469; PubMed Central PMCID: PMC4127108.
Citation index: 13
29. **Mosha JF**, Sturrock HJ, Greenwood B, Sutherland CJ, Gadalla NB, Atwal S, Hemelaar S, Brown JM, Drakeley C, Kibiki G, Bousema T, Chandramohan D, Gosling RD. Hot spot or not: a comparison of spatial statistical methods to predict prospective malaria infections. Malar J. 2014;13:53. doi: 10.1186/1475-2875-13-53. PubMed PMID: 24517452; PubMed Central PMCID: PMC3932034.
Citation index: 37

30. **Mosha JF**, Sturrock HJ, Brown JM, Hashim R, Kibiki G, Chandramohan D, Gosling RD. The independent effect of living in malaria hotspots on future malaria infection: an observational study from Misungwi, Tanzania. Malar J 2014;13:445. doi: 10.1186/1475-2875-13-445. PubMed PMID: 25413016; PubMed Central PMCID: PMC4255924.
    Citation index: 11
31. **Nankabirwa J**, Brooker SJ, Clarke SE, Fernando D, Gitonga CW, Schellenberg D, Greenwood B. Malaria in school-age children in Africa: an increasingly important challenge. Trop Med Int Health 2014;19:1294-309. doi: 10.1111/tmi.12374. Epub 2014 Aug 22. Review. PubMed PMID: 25145389; PubMed Central PMCID: PMC4285305.
    Citation index: 11
32. **Nankabirwa JI**, Wandera B, Amuge P, Kiwanuka N, Dorsey G, Rosenthal PJ, Brooker SJ, Staedke SG, Kamya MR. Impact of intermittent preventive treatment with dihydroartemisinin-piperaquine on malaria in Ugandan schoolchildren: a randomized, placebo-controlled trial. Clin Infect Dis 2014;58:1404-12. doi: 10.1093/cid/ciu150. Epub 2014 Mar 12. PubMed PMID: 24621953; PubMed Central PMCID: PMC4001293.
    Citation index: 26
33. **Ocholla H,** Preston MD, Mipando M, Jensen AT, Campino S, MacInnis B, Alcock D, Terlouw A, Zongo I, Oudraogo JB, Djimde AA, Assefa S, Doumbo OK, Borrmann S, Nzila A, Marsh K, Fairhurst RM, Nosten F, Anderson TJ, Kwiatkowski DP, Craig A, Clark TG, Montgomery J. Whole-genome scans provide evidence of adaptive evolution in Malawian Plasmodium falciparum isolates. J Infect Dis 2014;210:1991-2000. doi: 10.1093/infdis/jiu349. Epub 2014 Jun 19. PubMed PMID: 24948693; PubMed Central PMCID: PMC4241944.
    Citation index: 18
34. Preston MD, Assefa SA, **Ocholla H**, Sutherland CJ, Borrmann S, Nzila A, Michon P, Hien TT, Bousema T, Drakeley CJ, Zongo I, Ouédraogo JB, Djimde AA, Doumbo OK, Nosten F, Fairhurst RM, Conway DJ, Roper C, Clark TG. PlasmoView: a web-based resource to visualise global Plasmodium falciparum genomic variation. J Infect Dis 2014;209:1808-15. doi: 10.1093/infdis/jit812. Epub 2013 Dec 12. PubMed PMID: 24338354; PubMed Central PMCID: PMC4017360
    Citation index: 20
35. Preston MD, Campino S, Assefa SA, Echeverry DF, **Ocholla H,** Amambua-Ngwa A, Stewart LB, Conway DJ, Borrmann S, Michon P, Zongo I, Ouédraogo JB, Djimde AA, Doumbo OK, Nosten F, Pain A, Bousema T, Drakeley CJ, Fairhurst RM, Sutherland CJ, Roper C, Clark TG. A barcode of organellar genome polymorphisms identifies the geographic origin of Plasmodium falciparum strains. Nat Commun 2014;5:4052. doi: 10.1038/ncomms5052. PubMed PMID: 24923250; PubMed Central PMCID: PMC4082634.
    Citation index: 42

2015

36. Giorgi,E, **Sesay SSS**, Terlouw DJ, Diggle PJ. Combining data from multiple spatially referenced prevalence surveys using generalized linear geostatistical models. Stat Soc 2015; 178: 445-464. DOI:10.1111/rssa.12069
    Citation index: 23

37. **Matowo J**, Kitau J, Kaaya R, Kavishe R, Wright A, Kisinza W, Kleinschmidt I, Mosha F, Rowland M, Protopopoff N. Trends in the selection of insecticide resistance in Anopheles gambiae s.l. mosquitoes in northwest Tanzania during a community randomized trial of longlasting insecticidal nets and indoor residual spraying. Med Vet Entomol 2015;29:51-9. doi: 10.1111/mve.12090. Epub 2014 Dec 24. PubMed PMID: 25537754; PubMed Central PMCID: PMC4359020.
Citation index: 16
38. Nalwoga A, Cose S, Wakeham K, Miley W, **Ndibazza** J, Drakeley C, Elliott A, Whitby D, Newton R. Association between malaria exposure and Kaposi's sarcoma-associated herpes virus seropositivity in Uganda. Trop Med Int Health 2015;20:665-672. doi: 10.1111/tmi.12464. Epub 2015 Feb 9. PubMed PMID: 25611008; PubMed Central PMCID: PMC4390463.
Citation index: 11
39. **Nankabirwa JI**, Yeka A, Arinaitwe E, Kigozi R, Drakeley C, Kamya MR, Greenhouse B, Rosenthal PJ, Dorsey G, Staedke SG. Estimating malaria parasite prevalence from community surveys in Uganda: a comparison of microscopy, rapid diagnostic tests and polymerase chain reaction. Malar J 2015;14:528. doi: 10.1186/s12936-015-1056-x. PubMed PMID: 26714465; PubMed Central PMCID: PMC4696244.
Citation index: 15
40. **Ndiaye M,** Sylla K, Sow D, Tine R, Faye B, Ndiaye JL, Dieng Y, Lo AC, Abiola A, Cisse B, Ndiaye D, Theisen M, Gaye O, Alifrangis M. Potential Impact of Seasonal Malaria Chemoprevention on the Acquisition of Antibodies Against Glutamate-Rich Protein and Apical Membrane Antigen 1 in Children Living in Southern Senegal. Am J Trop Med Hyg 2015;93:798-800. doi: 10.4269/ajtmh.14-0808. Epub 2015 Aug 17. PubMed PMID: 26283746; PubMed Central PMCID: PMC4596602.
Citation index: 0
41. Samad H, Coll F, Preston MD, **Ocholla H,** Fairhurst RM, Clark TG. Imputation-based population genetics analysis of Plasmodium falciparum malaria parasites. PLoS Genet 2015 ;11:e1005131. doi: 10.1371/journal.pgen.1005131. eCollection 2015 Apr. PubMed PMID: 25928499; PubMed Central PMCID: PMC4415759.
Citation index: 10

2016

42. Awine T, Belko MM, Oduro AR, **Oyakhirome S**, Tagbor H, Chandramohan D, Milligan P, Cairns M, Greenwood B, Williams JE. The risk of malaria in Ghanaian infants born to women managed in pregnancy with intermittent screening and treatment for malaria or intermittent preventive treatment with sulfadoxine/pyrimethamine. Malar J 2016;15:46. doi: 10.1186/s12936-016-1094-z. PubMed PMID: 26821532; PubMed Central PMCID: PMC4730594.
Citation index: 2
43. **Kabula B,** Tungu P, Rippon EJ, Steen K, Kisinza W, Magesa S, Mosha F, Donnelly MJ. A significant association between deltamethrin resistance, Plasmodium falciparum infection and the Vgsc-1014S resistance mutation in Anopheles gambiae highlights the epidemiological importance of resistance markers. Malar J 2016;15:289. doi: 10.1186/s12936-016-1331-5. PubMed PMID: 27216484; PubMed Central PMCID: PMC4877992.

Citation index: 3

44. Kepha S, Nikolay B, Nuwaha F, Mwandawiro CS, **Nankabirwa J**, Ndibazza J, Cano J, Matoke-Muhia D, Pullan RL, Allen E, Halliday KE, Brooker SJ. Plasmodium falciparum parasitaemia and clinical malaria among school children living in a high transmission setting in western Kenya. Malar J 2016;15:157. doi: 10.1186/s12936-016-1176-y. PubMed PMID: 26969283; PubMed Central PMCID: PMC4788950.
 Citation index: 5
45. **Samb B**, Konate L, Irving H, Riveron JM, Dia I, Faye O, Wondji CS. Investigating molecular basis of lambda-cyhalothrin resistance in an Anopheles funestus population from Senegal. Parasit Vectors 2016;9:449. doi: 10.1186/s13071-016-1735-7. PubMed PMID: 27519696; PubMed Central PMCID: PMC4983014.
 Citation index: 4
46. **Nankabirwa JI,** Conrad MD, Legac J, Tukwasibwe S, Tumwebaze P, Wandera B, Brooker SJ, Staedke SG, Kamya MR, Nsobya SL, Dorsey G, Rosenthal PJ. Intermittent Preventive Treatment with Dihydroartemisinin-Piperaquine in Ugandan Schoolchildren Selects for Plasmodium falciparum Transporter Polymorphisms That Modify Drug Sensitivity. Antimicrob Agents Chemother 2016;60:5649-54. doi: 10.1128/AAC.00920-16. Print 2016 Oct. PubMed PMID: 27401569; PubMed Central PMCID: PMC5038325.
 Citation index: 5
47. Genomic epidemiology of artemisinin resistant malaria. MalariaGEN Plasmodium falciparum Community Project. Elife. 2016;5. pii: e08714. doi: 10.7554/eLife.08714. Collaborators: Amato R, Miotto O, Woodrow CJ, Almagro-Garcia J, Sinha I, Campino S, Mead D, Drury E, Kekre M, Sanders M, Amambua-Ngwa A, Amaratunga C, Amenga-Etego L, Andrianaranjaka V, Apinjoh T, Ashley E, Auburn S, Awandare GA, Baraka V, Barry A, Boni MF, Borrmann S, Bousema T, Branch O, Bull PC, Chotivanich K, Conway DJ, Craig A, Day NP, Djimdé A, Dolecek C, Dondorp AM, Drakeley C, Duffy P, Echeverry DF, Egwang TG, Fairhurst RM, Faiz MA, Fanello CI, Hien TT, Hodgson A, Imwong M, Ishengoma D, Lim P, Lon C, Marfurt J, Marsh K, Mayxay M, Michon P, Mobegi V, Mokuolu OA, Montgomery J, Mueller I, Kyaw MP, Newton PN, Nosten F, Noviyanti R, Nzila A, **Ocholla H**, Oduro A, Onyamboko M, Ouedraogo JB, Phyo AP, Plowe C, Price RN, Pukrittayakamee S, Randrianarivelojosia M, Ringwald P, Ruiz L, Saunders D, Shayo A, Siba P, Takala-Harrison S, Thanh TN, Thathy V, Verra F, Wendler J, White NJ, Ye H, Cornelius VJ, Giacomantonio R, Muddyman D, Henrichs C, Malangone C, Jyothi D, Pearson RD, Rayner JC, McVean G, Rockett KA, Miles A, Vauterin P, Jeffery B, Manske M, Stalker J, MacInnis B, Kwiatkowski DP.
 Citation index: Not found

2017

48. **Ndiaye M**, Sow D, Nag S, KSylla K, Tine RC, J Ndiaye JL, Lo AC, Gaye O, Fay B, Alifrangis M. Country-Wide Surveillance of Molecular Markers of Antimalarial Drug Resistance In Senegal by Use of Positive Malaria Rapid Diagnostic Tests. Am J Trop Med Hyg 2017;97:1593-1596. Doi: 10.4269/ajtmh.17-0021

49. **Osarfo J,** Tagbor H, Cairns M, Alifrangis M, Magnussen P. Dihydroartemisinin-piperaquine versus artesunate-amodiaquine for treatment of malaria infection in pregnancy in Ghana: an open-

label, randomised, non-inferiority trial. Trop Med Int Health 2017;22:1043-1052. doi: 10.1111/tmi.12905. Epub 2017.

50. **Sesay SSS**, Giorgi E, Diggle PJ, Schellenberg D, LallooD, Terlouw DJ. Surveillance in easy to access population subgroups as a tool for evaluating malaria control progress: A systematic review. PLoS One2017;12:e0183330.

51. **Tine RC**, Sylla K, Faye BT, Poirot E, Fall FB, Sow D, Wang D, Ndiaye M, Ndiaye JL, Faye B, Greenwood B, Gaye O, Milligan P. Safety and Efficacy of Adding a single Low Dose of Primaquine to the Treatment of Adult Patients With Plasmodium falciparum Malaria in Senegal, to Reduce Gametocyte Carriage: A Randomized Controlled Trial. Clin Infect Dis 2017; 65:535-543. doi: 10.1093/cid/cix355. PubMed PMID: 28605472.
